# Does dissemination extend beyond publication: a survey of a cross section of public funded research in the UK

**DOI:** 10.1186/1748-5908-5-61

**Published:** 2010-08-04

**Authors:** Paul M Wilson, Mark Petticrew, Michael W Calnan, Irwin Nazareth

**Affiliations:** 1 Centre for Reviews and Dissemination, University of York, YO10 5DD, UK; 2 Public and Environmental Health Research Unit, London School of Hygiene and Tropical Medicine, WC1E 7HT, UK; 3 School of Social Policy, Sociology and Social Research, University of Kent, CT2 7NF, UK; 4 MRC General Practice Research Framework, University College London, NW1 2ND, UK

## Abstract

**Background:**

In the UK, most funding bodies now expect a commitment or effort on the part of grant holders to disseminate the findings of their research. The emphasis is on ensuring that publicly funded research is made available, can be used to support decision making, and ultimately improve the quality and delivery of healthcare provided. In this study, we aimed to describe the dissemination practices and impacts of applied and public health researchers working across the UK.

**Methods:**

We conducted a survey of 485 UK-based principal investigators of publicly funded applied and public health research. Participants were contacted by email and invited to complete an online questionnaire via an embedded URL. Gift vouchers were given to all participants who completed the questionnaire. Four reminder emails were sent out to non-respondents at one, two, three, and four weeks; a fifth postal reminder was also undertaken.

**Results:**

A total of 243/485 (50%) questionnaires were returned (232 completed, 11 declining to participate). Most researchers recognise the importance of and appear committed to research dissemination. However, most dissemination activity beyond the publishing of academic papers appears to be undertaken an *ad hoc *fashion. There is some evidence that access to dissemination advice and support may facilitate more policy interactions; though access to such resources is lacking at an institutional level, and advice from funders can be variable. Although a minority of respondents routinely record details about the impact of their research, when asked about impact in relation to specific research projects most were able to provide simple narrative descriptions.

**Conclusions:**

Researchers recognise the importance of and appear committed to disseminating the findings of their work. Although researchers are focussed on academic publication, a range of dissemination activities are being applied albeit in an *ad hoc *fashion. However, what constitutes effective dissemination (in terms of impact and return on investment) remains unclear. Researchers need greater and clearer guidance on how best to plan, resource, and facilitate their dissemination activities.

## Background

There is renewed interest and emphasis on the gap between research and policy and practice, both nationally in the UK and internationally. In the UK, a series of policy reviews have highlighted gaps and suggested measures to enhance the uptake of research knowledge into routine clinical practice[1-3]. The Government's 2006 health research strategy also set out a range of related objectives that include improving the nation's health and wealth, delivering value for money, and ensuring that research knowledge is made readily available to health professionals, researchers, and the public[4]. Internationally, the WHO has recommended that stronger emphasis should be placed on translating knowledge into actions to improve health, essential if the health-related Millennium Development Goals are to be achieved by 2015[5].

The emphasis on research to practice is reflected in the levels of support for dissemination among those public agencies funding applied and public health research. A recent study has shed light on the efforts of research funders to support and promote dissemination activity[6]. However, this found that whilst funders are engaged in a range of activities, there appears to be some lack of clarity among funding agencies as to what is meant by, and in the degree to which they themselves engage in, dissemination activity. In addition, although a majority of funders considered dissemination to be a shared responsibility, there was variation in their expectations of the role and contribution to be made by researchers. Given these findings, it seems appropriate to shed light on the role, views, and practices of researchers in relation to dissemination.

Throughout this study we have defined and used the term dissemination (a subset of knowledge translation) to describe a planned process that involves consideration of target audiences and the settings in which research findings are to be received and, where appropriate, communicating and interacting with wider policy and health service audiences in ways that will facilitate research uptake in decision making processes and practice.

This study focuses on the role of the researcher in the dissemination of applied and public health research. In the UK, most funding bodies now expect efforts on the part of grant holders to disseminate the findings of their research. In addition, most researchers are aware that the National Institute for Health Research (NIHR) is seeking to maximise the impact of its £800 million investment in applied health research[7]. The NIHR has expectations that researchers will work to ensure that research is made available, can be used to support decision making, and ultimately improve the quality and delivery of healthcare provided.

Against this background, we aimed to describe how public health and health services researchers working across the UK disseminate the findings of their research. We also sought to determine:

1. whether we could identify any explicit/implicit use of existing knowledge and theory relating to research dissemination;

2. whether researchers knew of or could describe any impacts their activities had had on health policy and practice;

3. whether we could identify any dissemination factors associated with respondents ability to report research impacts.

## Methods

We conducted a survey to explore how researchers disseminate the findings of their research. We sought to elicit grant holder views on: the purpose of dissemination; their own current practices; their perceived role/responsibility for dissemination and that of the funders; perceived successes and failures; potential areas for improvement; and evaluation of and reflection on wider impact.

### Survey Instrument

The online questionnaire was based on an instrument that was piloted with, and administered to, senior researchers in intramural MRC research units[8]. Question development was informed by a systematic review of dissemination planning frameworks and strategies (Wilson PM, Petticrew M, Calnan MW, Nazareth I: Dissemination: researchers should do what? A systematic review of conceptual planning frameworks, Submitted). Many of the identified frameworks share common theoretical underpinnings and propose that the effectiveness of dissemination is influenced by due consideration of a number of key elements. These include planning activities, targeting audiences, selecting communication channels, and evaluating impact. Using these elements as an underpinning framework, we devised an instrument that comprised a series of 36 open and closed questions (see Additional File 1).

The first part of the questionnaire was designed to elicit researcher views and attitudes on the dissemination of research and to capture descriptions of their practices generally. The second part of the questionnaire asked respondents to think about a particular grant rather than just their activities in general and included specific questions designed to capture any research impacts on health policy, clinical guideline development, or on the organisation and or delivery of healthcare and services. The research impact questions were based on a recently developed research impact framework that presents a structure for capturing narrative descriptions of research impact[9,10]. Using the themes of this framework, we devised four open-ended questions intended to capture self-reported descriptions of research impacts. Respondents were asked to answer these questions in relation to the dissemination of a publicly funded research project they had recently completed. The final online questionnaire could be completed in around 30 minutes.

### Survey sample

Ten UK funding agencies were contacted in July 2008 and invited to provide (secure and encrypted) email contact details for UK-based principal investigators of applied health services and public health research completed in between 2003 and 2008. Five agencies (Chief Scientist Office, Economic and Social Research Council, Medical Research Council, NIHR Health Technology Assessment Programme, and Wellcome Trust) responded and provided details. Principal investigator details for one non-responding agency (NIHR Service Delivery and Organisation Programme) were obtained from their website. Two agencies (British Heart Foundation and Joseph Rowntree Foundation) indicated that they funded very little public health and applied health services research, and so were excluded from the survey. The Department of Health Policy Research Programme and Cancer Research UK responded stating that they were unable provide details of principal investigators.

Email addresses were sourced for the 743 principal investigators identified. The complete list was then de-duplicated resulting in a total survey sample of 536 potential participants.

### Survey administration

On 13 October 2008, all participants were contacted by email, told the purpose of the study, and invited to complete an online questionnaire via an embedded URL. The online questionnaire was hosted by SurveyMonkey website http://www.surveymonkey.com. As part of the study, we nested a randomised element to test the utility of offering an incentive. Participants were randomly allocated to receive either 'knowledge of' or 'no knowledge of' a £10 Amazon gift voucher. The gift vouchers were given to all participants who completed the questionnaire regardless of randomisation. Full details of the randomised element of this study are reported elsewhere[11].

Reminder emails were sent out to non-respondents at one, two, three, and four weeks. A paper version of the questionnaire was posted out as a fifth reminder.

A combination of IP address and questionnaire responses were used to identify multiple responses from a single participant[12]. Where multiple responses from a single participant occurred, the most recently completed questionnaire was retained for analysis. Non-invited responses from respondents out with the study sample were excluded from the analysis.

Data derived from the survey questionnaires were analysed in SPSS version 15.0 (SPSS Inc, Chicago, IL.). The free text derived from the open-ended questions was coded, grouped, and themes identified. Ethical approval for the study was obtained from the University of York IRISS Ethics Committee.

## Results

Of the 536 identified email addresses, 51 were undeliverable leaving a revised sample of 485. A total 243 questionnaires were returned (232 completed, 11 declining to participate) giving a response rate of 50%. Four questionnaires were completed by non-invited individuals, and these were excluded from the analysis. Two participants submitted multiple responses; the most recently submitted questionnaire was retained for analysis in each case. Any questionnaires not returned by 31 December 2008 were deemed to be non-responses.

### Importance of dissemination

Research dissemination was rated as important or very important by 216 (93%) respondents, all of whom thought that it was part of their role as a researcher. Only two respondents felt that research dissemination was not important to their own research. Table 1 shows the reasons selected (from a predefined list of 12 options) by respondents for disseminating the findings of their research. Of the 26 respondents who provided additional reasons, eight stated said they undertook dissemination for professional career advancement purposes, seven saw it as a way of providing feedback to study participants, two did it to move the research agenda forward and two stated it was ethical to do so. Of the reasons given, one-third (n = 78) of respondents indicated that raising awareness was the most important reason, followed by those who felt influencing practice (n = 43) and policy (n = 40) were the most important reasons.

**Table 1:** Reasons for disseminating the findings of research

Reason	N (%)
Raise awareness of findings	216 (93)
Influence policy	198 (85)
Influence practice	195 (84)
Stimulate discussion/debate	173 (75)
Transfer research to practice	173 (75)
Raise the organisational profile	150 (65)
Attract future funding	145 (62)
Research Assessment Exercise	123 (53)
Justify public funding	112 (48)
Promote public understanding of science	93 (40)
Satisfy contractual requirements	85 (37)
Improve your own communication	56 (24)
Other	26 (11)

### Resources available for dissemination

Forty-seven (20%) respondents stated that they had a dedicated person or team responsible for dissemination-related activities within their unit or department. Of these, 20 stated that this entailed access to departmental or institutional communications support, and three indicated that there was a member of the research team with specific skills. Two-thirds of respondents (n = 151) estimated that the proportion of their time that they dedicate to dissemination-related activities was less than 10% (one-half day per week).

### Planning and targeting dissemination activity

Forty-six (20%) respondents indicated that their unit or department had a formal communication/dissemination strategy. Twenty (9%) respondents stated that they usually referred to guidance or used a framework to plan their dissemination activity; a further 51 (22%) said that they sometimes did so.

Three-quarters of respondents (n = 170) indicated that as part of research dissemination activities they would consider how audiences or groups would like to access, read, and use research findings. Only one respondent stated that they would never think about this. Most respondents (n = 195) also indicated that part of their dissemination planning involved considering whether to target specific audiences (such as policy makers, service managers, or general practitioners). Just under one-third (n = 71) stated that they would then go on to produce a research summary or key messages that were written for specific audiences.

Table 2 shows the communication channels utilised by respondents. Most of the responses in the 'other' category were already covered by the other items listed in Table 2. The additional channels included dissemination via community groups and charities (n = 4), via training packages (n = 3), via DVDs (n = 1), and via posters in general practices (n = 1). Of the channels utilised, just over one-half (n = 129) felt that dissemination via academic journals generally had the most impact.

**Table 2:** Communication channels utilised by researchers

Communication channel	N (%)
Academic journal	227 (98)
Conference presentation	223 (96)
Report to funder	212 (91)
Seminar	145 (62)
Workshop	133 (57)
Web access to full report	125 (54)
Web access to summary report	116 (50)
Issued press release	112 (48)
Networking	106 (46)
Face to face meetings	93 (40)
Newsletter	91 (39)
Media interview	74 (32)
Research Register	74 (32)
Policy Briefing paper	41 (17)
Targeted mailings	38 (16)
Email alerts	18 (8)
CD-Rom	6 (3)
RSS feeds	1 (<1)
Other	34 (15)

### Evaluating impact

Respondents were asked how they recorded formal or informal feedback about the impact of their research. Around one-half (n = 115) stated that such information is not formally recorded, with a further one-quarter (n = 59) saying that it is written down for personal use. Only eleven respondents stored this type of information in a database, with a further nine storing with paper project files or collating into an annual report. The remainder were either not sure or did not answer the question. Thirty (13%) respondents stated that they usually evaluated the success/impact of research dissemination activities.

Overall, most respondents (84%, n = 194) rated their current research dissemination activities as either good or adequate; only two rated them as excellent. A further 10% (n = 23) rated their current activities as poor, while eight others were not sure. The remainder did not answer the question.

### Capturing research impacts

For the remaining questions, respondents were asked to provide information on the dissemination of a publicly funded research project they had recently completed; 95% (n = 220) provided some detail.

Around two-thirds (65%, n = 150) of respondents indicated that a dissemination plan was produced for the research project. Table 3 shows which funding agencies also provided advice or support. The type of support and advice from funders varied and included: advice on structure, length, content, and style of final report and related outputs (n = 39); press office/release support (n = 8); media training (n = 2); financial support for stakeholder workshops and meetings (n = 5); facilitating meetings with relevant policy makers (n = 2); the production of patient/lay information leaflets (n = 3).

**Table 3:** Dissemination advice or support provided by funding agencies?

Funder	Yes	No	Not sure	Total
British Heart Foundation	0	2	0	2
Cancer Research UK	0	2	0	2
Chief Scientist Office	9	20	2	31
Department of Health Policy Research Programme	2	3	0	5
Economic and Social Research Council	7	4	3	14
Medical Research Council	9	17	3	29
NIHR Health Technology Assessment Programme	23	26	6	55
NIHR Service Delivery and Organisation Programme	15	8	0	23
Wellcome Trust	1	5	0	6
Other	20	25	8	53
Total	86	112	22	220

Researchers were also asked whether they knew of or could describe any impacts their activities had had on health policy and practice; 70% (n = 162) were able to provide some detail. Just over one-half (51%, n = 120) indicated that their research had led to discussions or interactions with policy makers and or been cited or included in policy documents. The interactions included direct engagement with national and international government ministers, local NHS commissioning agencies, National Institute of Health and Clinical Excellence (NICE) Appraisal Committees (and European equivalents), the National Screening Committee, Parliamentary Select Committee for Health, and the National Clinical Directors for Cancer and Mental Health.

Sixty-seven (28%) respondents stated that their research had been cited in clinical guidelines; one-half of whom stated their findings were cited as part of guidance issued by NICE. Around one-half (49%, n = 114) of respondents stated that their research had, or was likely to have, influence on the acceptability and or availability of a health intervention(s) or on the organisation and delivery of health services. Around one-third (n = 44) of these pointed to their work being incorporated into clinical guidelines as proxy evidence of influence. Others named specific interventions or services in which they anticipated accelerated and enhanced provision or disinvestment as likely consequences of their research.

Twenty-nine respondents indicated that they felt the findings of their research had been misrepresented or used in ways that they felt were inappropriate; 15 of which referred to misrepresentation in the mass media.

Table 4 explores whether the respondents reporting of impacts was associated with the amount of time that was devoted to dissemination, whether activity was planned, whether they had access to departmental support, or advice and support from funders. We also looked at whether reporting of impacts was associated with the view that academic publication generates the most impact. Those respondents receiving dissemination advice and support and/or who believe that researchers need to do more than publish academic journal articles were more likely to report policy impacts. In addition, having access to dissemination support, be it departmental or from the funder, appears to increase the chance that research findings are misreported. None of the other factors were statistically significant.

**Table 4:** Dissemination factors associated with the reporting of research impacts

Time spent on dissemination	>10%	<10%	X^2^
Reported policy impacts	38/69	80/145	p = 0.98
Reported health service impacts	28/67	85/145	p = 0.02
Reported cited in clinical guidelines	18/68	48/144	p = 0.31
Reported misreporting	14/68	14/147	p = 0.02
			
**Dissemination plan produced by researchers**	**Yes**	**No**	**X^2^**
Reported policy impacts	81/145	39/120	p = 0.52
Reported health service impacts	72/142	42/71	p = 0.24
Reported cited in clinical guidelines	47/143	20/70	p = 0.52
Reported misreporting	20/146	9/71	p = 0.83
			
**Dissemination support within dept**	**Yes**	**No**	**X^2^**
Reported policy impacts	31/46	89/170	p = 0.06
Reported health service impacts	26/46	88/168	p = 0.62
Reported cited in clinical guidelines	17/46	50/168	p = 0.35
Reported misreporting	13/45	16/172	p < 0.01
			
**Advice/support received from funder**	**Yes**	**No**	**X^2^**
Reported policy impacts	62/83	58/132	p < 0.01
Reported health service impacts	45/83	69/130	p = 0.87
Reported cited in clinical guidelines	28/81	39/132	p = 0.44
Reported misreporting	18/85	11/121	p < 0.01
			
**Believe that academic journals have most impact**	**Yes**	**No**	**X^2^**
Reported policy impacts	61/124	51/73	p < 0.01
Reported health service impacts	63/123	39/72	p = 0.69
Reported cited in clinical guidelines	41/123	19/73	p = 0.28
Reported misreporting	17/125	9/73	p = 0.79

Respondents were asked whether there were any methods of disseminating research findings that they would like to have used but are unable to do so; just under one-fifth (18%, n = 42) said yes. These included better access to policy makers (n = 6), media coverage (n = 8), e-dissemination (n = 6), workshops (n = 3), funds for open access publishing (n = 2), and materials for participant feedback (n = 3).

Respondents were asked if there was anything else that would have enhanced the impact of this research; 29% (n = 68) said yes. Eleven respondents thought greater funding for dissemination would have led to greater impact. To illustrate, one suggested that it was difficult to undertake dissemination when staff had to move onto other projects. Another suggested the establishment of a central funding pool that could, when appropriate, be called on for more dissemination activity and to pay something towards the staff time involved. Others suggested: greater targeting and face-to-face engagement with policy makers (n = 10); more targeted dissemination to key stakeholders and relevant front line audiences (n = 11); the need for protected time to write and prepare journal articles and final reports (n = 7) and that findings are published in a more timely fashion (n = 4); more enthusiasm and active engagement from the funders beyond simply publishing the report (n = 5). Five respondents would have liked to have had more support to develop websites and podcasts, and a further six stated that with more time and support to respond to media interest, the impact of their research would have been greater.

Respondents were also asked whether the findings of their research had been taken up or used by anyone that they hadn't anticipated or in any other ways that were not originally anticipated; 15% (n = 36) said yes. Nine respondents indicated that international interest and uptake had been greater than originally anticipated.

## Discussion

### Principal findings

This survey presents an overview of the ways by which health services researchers working across the UK are disseminating the findings of their research. Although we are aware of studies that explore the nature of dissemination activity in other countries[13,14], we are unaware of any previous survey that describes dissemination activities in the UK. Given this, the findings of this study will provide a baseline against which future dissemination developments can be measured.

It appears many researchers recognise the importance of, and appear committed to, research dissemination. Perhaps unsurprisingly, for a population undertaking applied health research, respondents appear motivated by a desire that their research influences and ultimately helps improve health outcomes and the quality of healthcare delivered. This motivation may also explain why a majority knew of, and could easily describe, the impacts their research had had on policy and practice. This, despite of the fact that one-half of respondents said they do not formally record such impact information.

In this survey, the methods researchers employ to disseminate research findings are focussed on the traditional academic outputs of peer reviewed research papers and conference presentations. Other push, pull, and 'linkage and exchange' elements[15,16] are utilised, but in an *ad hoc *and opportunistic fashion. Nevertheless, researchers do appear to have some awareness and understanding of dissemination theory, in particular respondents recognised the need to plan, identify, and target key messages at specific audiences. Although most would routinely think about targeting potential end users, only one-third would actually do so in practice.

There is some suggestion in this survey that access to dissemination advice and support and undertaking activity that goes beyond publishing academic journal articles may generate more policy interactions. However, most respondents indicated that access to such advice and support was lacking at an institutional level, and that the nature of provision by funders appears to be variable.

### Strengths and weaknesses

Our survey achieved a response rate of 50%. By way of contrast, a recent survey by the Research Information Network and Joint Information Systems Committee exploring the influence of the Research Assessment Exercise (RAE) on the dissemination behaviours and attitudes of 8,000 researchers from different subjects and disciplines achieved a response rate of 25%[17]. Those responding to our survey clearly view research dissemination as highly relevant to their work, so it is possible that the value and importance may be overestimated. Nor can we entirely rule out the possibility of social desirability bias on the part of respondents -- telling us what they think we want to hear. Given this, we suggest some caution in generalising these findings to the research population as a whole.

In this survey, a 36-item online questionnaire was utilised; we adhered to suggested recommendations of good practice for the design of email questionnaires[18,19]. However, we recognise that shorter postal questionnaires are associated with increased response rates[18]. It may also be that the incentive offered for time invested was deemed inadequate leverage by some participants, especially if considered in relation to their paid salary as professional researchers. In addition, receipt of the incentive was dependant on questionnaire completion, and it may be that a higher response rate may have been possible had the incentive been given upfront, unconditionally.

There is an increasing requirement, particularly of publicly funded research, for some measure of impact or evaluation. There are a number of specialised research impact assessment approaches, but these usually require specialist skills and additional resources[20,21]. Our decision to adopt and adapt a more pragmatic framework[9,10] and ask for simple narrative descriptions for impacts on health policy and practice appears justified. Although only a minority indicated that they routinely recorded formal or informal feedback about the impact of their research, when asked about impact in relation to specific research they had recently completed most respondents were able to provide examples. This is an approach to recording research impacts that merits further consideration by funders, researchers, and their institutions alike.

### What this study adds

The focus on the traditional academic mediums of journals and conference presentations and *ad hoc *use of other available communication channels was also found in our pilot of intramural MRC research units, where many indicated that undertaking such activity can be difficult as knowledge translation can often go unfunded[8]. The focus on academic publication is somewhat unsurprising given the emphasis of the 2008 UK RAE (the process by which higher education funding bodies determine the level of research funding they provide to UK universities). In the RAE, the key indicator of research excellence was publication in high impact scientific journal rather than actual or anticipated impacts on health policy and practice. A much greater emphasis on research-driven impacts to the economy, society, policy, and quality of life is proposed for the future Research Excellence Framework, but traditional academic output will continue to dominate and are expected to contribute around 60% of the overall assessment outcome[22].

Most respondents thought that publication in high-impact academic journals generally has the most impact. Several respondents indicated that this was due in part to the media coverage often generated by such publications; the inference being that coverage was generated by the 'push' efforts of the journal. Media engagement appears to be common, with around one-half of researchers indicated that they routinely issue press releases. There is a suggestion having some access to dissemination support, be it departmental or from the funder, may increase the chance that research findings are misreported; one-half of the examples of misreporting related to mass media representations. Media engagement can be an effective method of raising awareness, but researchers should recognise that there will be a trade off between media coverage and the perceived accuracy of media reports generated. Given levels of media engagement are quite high, it may be that researchers need to be more aware of potential costs and benefits associated with this approach, and need to engage more with the third-party media dissemination that is undertaken on their behalf.

In this survey, there is some suggestion that the lack of clarity apparent among funding agencies around what constitutes knowledge translation[6] may also extend to individual researchers. This lack of clarity may partly explain why some respondents indicated they did not receive any dissemination advice and support from specific funders whilst others said they did (Table 3). For example, of those stating that they had received advice and support from the funder, around one-half indicated that this was advice on structure and style of a final report rather than on the appropriateness of their plans for dissemination. It may be that that those reporting no support have a clearer understanding of the differences between what constitutes publication and what constitutes dissemination.

We have previously raised concerns about the nature and type of guidance issued by funding bodies to their grant holders and applicants[23]. Although there are a number of theoretically informed frameworks available that could be used by researchers to help guide their dissemination planning and activity (Wilson PM, Petticrew M, Calnan MW, Nazareth I: Dissemination: researchers should do what? A systematic review of conceptual planning frameworks, Submitted), UK funding bodies do not appear to be providing much in the way of dissemination guidance to their grant holders. Despite this and although the evidence is somewhat limited, policy interactions did appear to be associated with funder involvement, suggesting that funders are often best placed to facilitate introductions and engagement.

Researchers need clearer guidance on how best to plan, resource, and facilitate their dissemination activity. UK funders are well placed to influence this activity. Given the current emphasis on reducing the 'gaps in translation' and on the need to deliver tangible returns on the substantial investment in applied health research, funders should be encouraging their grant holders to adopt a more structured and theoretically informed approach to their research dissemination at the grant application stage. A structured approach would identify upfront any potential resource implications, provide greater clarity on (and engagement with) the end user, and hopefully deliver more efficient and appropriate research communications. Such an approach would also provide an opportunity to drive the science of knowledge translation forward, providing opportunities to rigorously evaluate whether taking a more theoretically informed approach or investing more time and effort on research dissemination does enhance the uptake of research findings in policy and practice.

## Summary

Researchers recognise the importance of, and appear committed to, disseminating the findings of their work. Although researchers are unsurprisingly focussed on academic publication, a range of dissemination activities is being applied, albeit in an *ad hoc *fashion. However, what constitutes effective dissemination (in terms of impact and return on investment) remains unclear. Researchers need greater and clearer guidance on how best to plan, resource, and facilitate their dissemination activities.

## Competing interests

Paul Wilson is an Associate Editor of Implementation Science. All decisions on this manuscript were made by another senior editor. The author(s) declare that they have no other competing interests.

## Authors' contributions

All authors contributed to the conception, design, and analysis of the study. PMW led the data collection and analysis for the study. All authors were involved in the writing of the first and subsequent versions of the paper, and read and approved the final manuscript. PMW is the guarantor of the study.

## Supplementary Material

Additional file 1 **Does dissemination extend beyond publication: survey instrument**. Paper version of the online questionnaire. Click here for file
