# Supplementary material for: Does dissemination extend beyond publication: a survey of a cross section of public funded research in the UK
Source: Implement Sci. 2010 Aug 4;5:61. doi: 10.1186/1748-5908-5-61 (PMC2922079; doi:10.1186/1748-5908-5-61)
Supplement: Additional file 1 — Does dissemination extend beyond publication: survey instrument. Paper version of the online questionnaire. [file 1748-5908-5-61-S1.DOC]

**Disseminating the findings of health services and public health research**

This survey aims to find out what steps public health and health services researchers working across the UK are currently taking to disseminate the findings of their research.

For this project, we are defining dissemination as an active and planned process that involves ensuring that those who need to know about a piece of research get to know about it and can make sense of the findings.

The questionnaire contains 36 questions and can be completed in about 30 minutes.

Any information provided will be treated in the strictest confidence and presented on a non-attributed basis.

Please do not circulate to other colleagues.

The survey is part of a three-year project funded by the MRC Population Health Sciences Research Network (Knowledge translation to support the dissemination and implementation of MRC research on public health and health services policy. Ref: PHSRN 11). The project aims to identify ways by which the uptake of publicly funded clinical and public health and health services research can be enhanced.

The project team comprises: Mark Petticrew, London School of Hygiene and Tropical Medicine; Paul Wilson, Centre for Reviews and Dissemination; Mike Calnan, University of Kent; and Irwin Nazareth, MRC General Practice Research Framework.

For more details, contact Paul Wilson (pmw7@york.ac.uk).

**1. Please enter your name:**

|  |
| --- |

**2. Please enter your email**

|  |
| --- |

**3. Is the dissemination of research findings formally part of your role?**

Yes ❑

No ❑

**4. Do you think the dissemination of research findings should be formally part of your role?**

Yes ❑

No ❑

Not sure ❑

**5. How important to your own research is the process of dissemination?**

Very important ❑

Important ❑

Somewhat important ❑

Not important ❑

Not sure ❑

**6. How important is the process of research dissemination to the work of your unit/ department?**

Very important ❑

Important ❑

Somewhat important ❑

Not important ❑

Not sure ❑

**7. Is there a dedicated person or team responsible for dissemination related activities within your unit/organisation?**

No ❑

Not sure ❑

Yes ❑

If yes, please give details:

|  |
| --- |

**8. Can you estimate the proportion of your own time that is dedicated to dissemination related activities?**

None ❑

Less than 5% (*i.e.*, less than two hours a week) ❑

Between 5 and 10% ❑

Between 10 and 20% ❑

Between 20 and 30% ❑

Between 30 and 40% ❑

Between 40 and 50% ❑

More than 50% ❑

**9. Why do you disseminate the findings of your research?**

Please tick all that apply

To raise awareness of the findings ❑

To stimulate discussion/ debate ❑

To influence policy ❑

To influence practice ❑

To transfer research to practice ❑

To justify public funding ❑

To attract future funding ❑

To raise the organisational profile ❑

For the Research Assessment Exercise ❑

To improve your own communication ❑

To promote public understanding of science ❑

To satisfy contractual obligations ❑

Other (please give details) ❑

|  |
| --- |

**10. Which of the reasons given for disseminating the findings of your research are the most important?**

Most important ____________________________________

2^nd^ most important ____________________________________

3^rd^ most important ____________________________________

**11. Does your unit/ department have a formal communication/ dissemination strategy?**

Yes ❑

No ❑

Not sure ❑

**12. Do you ever refer to guidance or use a framework to plan dissemination-related activities?**

Always ❑

Usually ❑

Sometimes ❑

Rarely ❑

Never ❑

Not sure ❑

**13. At what stage in the research process do you usually plan dissemination-related activities?**

When the research is being formulated ❑

At the proposal stage ❑

During the research process ❑

At the draft report stage ❑

At the final report stage ❑

At all stages of the process ❑

**14. As part of your research dissemination, do you ever think about who needs to know about the findings and/or who is most likely to be influenced or will influence others?**

Always ❑

Usually ❑

Sometimes ❑

Rarely ❑

Never ❑



**15. As part of your research dissemination, do you ever consider how audiences or groups you would like to reach access, read, and use research findings?**

Always ❑

Usually ❑

Sometimes ❑

Rarely ❑

Never ❑

**16. What methods do you usually use to disseminate research findings?**

**Please tick all that apply**

Academic journals (*e.g.*, BMJ) ❑

Professional journals (*e.g.*, Pulse) ❑

Report to funders ❑

Full report (paper) ❑

Full report (web access) ❑

Summary report (paper) ❑

Summary report (web access) ❑

Press releases ❑

Newsletters ❑

Policy briefing paper ❑

Email alerts ❑

RSS feeds ❑

Targeted mailings ❑

Academic conferences ❑

Other conferences ❑

Seminars ❑

Workshops ❑

Face to face meetings ❑

Networking ❑

Media interviews ❑

Research registers ❑

CD-ROMs ❑

Other (please give details)

|  |
| --- |

**17.** **Of the methods you use to publish and disseminate the research findings, which do you think generally have the most impact?**

|  |
| --- |

**18. Do you ever produce research summaries or key messages that are written for specific audiences or groups (such as policy makers, service managers, or general practitioners)?**

Always ❑

Usually ❑

Sometimes ❑

Rarely ❑

Never ❑

**19. Do you ever evaluate the impact of your research?**

Always ❑

Usually ❑

Sometimes ❑

Rarely ❑

Never ❑

**20. Overall, how do you rate your current research dissemination activities?**

Excellent ❑

Good ❑

Adequate ❑

Poor ❑

Not sure ❑

**For the remaining questions, we would like you think about a publicly funded research project you have recently completed.**

**21. What was the title of the research project?**

|  |
| --- |

**22. Please state who funded the research project**

|  |
| --- |

**23. Was a dissemination plan produced for the research project?**

Yes ❑

No ❑

Not sure ❑

**24. Did you receive any advice or support from the funders?**

No ❑

Not sure ❑

Yes ❑

If yes, please give details:

|  |
| --- |

**25. What methods were used to publish and disseminate the findings of the research project ?**

**Please tick all that apply**

Academic journals (*e.g.*, BMJ) ❑

Professional journals (*e.g.*, Pulse) ❑

Report to funders ❑

Full report (paper) ❑

Full report (web access) ❑

Summary report (paper) ❑

Summary report (web access) ❑

Press releases ❑

Newsletters ❑

Policy briefing paper ❑

Email alerts ❑

RSS feeds ❑

Targeted mailings ❑

Academic conferences ❑

Other conferences ❑

Seminars ❑

Workshops ❑

Face to face meetings ❑

Networking ❑

Media interviews ❑

Research registers ❑

CD-ROMs ❑

Other (please give details)

|  |
| --- |

**26.** **Of the methods you used to publish and disseminate the research findings, which do you think had the most impact?**

|  |
| --- |

**27. Were there any methods of disseminating research findings that you would like to have used but are unable to do so?**

No ❑

Not sure ❑

Yes ❑

If yes, please give details:

|  |
| --- |

**28. Is there anything else you can think of that would have enhanced the impact of this research?**

No ❑

Not sure ❑

Yes ❑

If yes, please give details:

|  |
| --- |

**29. Have the findings of this research project been cited in clinical guidelines?**

No ❑

Not sure ❑

Yes ❑

If yes, please give details:

|  |
| --- |

**30. Has this research led to any discussions or interactions with policy makers, been cited or included in policy documents, or is likely to have any other influences on policy?**

No ❑

Not sure ❑

Yes ❑

If yes, please give details:

|  |
| --- |

**31. Has this research had, or is likely to have any influence on the acceptability and or availability of a health intervention(s) or on the organisation and delivery of health services.**

No ❑

Not sure ❑

Yes ❑

If yes, please give details:

|  |
| --- |

**32.** **Was this research disseminated by anyone else other than yourself (and the other investigators)?**

No ❑

Not sure ❑

Yes ❑

If yes, please give details:

|  |
| --- |

**33. Has this research been taken up or used by anyone that you didn't anticipate or in any other ways that were not originally anticipated?**

No ❑

Not sure ❑

Yes ❑

If yes, please give details:

|  |
| --- |

**34. Have the findings of this research ever been misrepresented or used in ways that you feel were inappropriate?**

No ❑

Not sure ❑

Yes ❑

If yes, please give details:

|  |
| --- |

**35. When you receive formal or informal feedback about the impact of your research how is this information recorded?**

It’s not formally recorded ❑

It’s written down for personal use ❑

It’s entered into a database ❑

Not sure ❑

Other (please specify) ❑

|  |
| --- |

**36. We would like to conduct some follow-up interviews to find out more about the nature of dissemination activities currently being utilised by UK researchers.**

As a token of our appreciation, interviewees will receive an £20 Amazon gift voucher.

Please indicate if you would be willing to take part in such an interview:

No ❑

Yes ❑

**Thank you for taking the time to complete this questionnaire**
